# Supplementary material for: Exploring the value in variations of the Relative Income Price (RIP) for calculating cigarette affordability: An illustration using Malaysia
Source: PLoS One. 2024 Nov 15;19(11):e0313695. doi: 10.1371/journal.pone.0313695 (PMC11567636; doi:10.1371/journal.pone.0313695)
Supplement: S1 Table — (DOCX) [file pone.0313695.s001.docx]

**Supporting Information to accompany “*Exploring the Value in Variations of the Relative Income Price (RIP) for Calculating Cigarette Affordability: An Illustration using Malaysia*”**

| **Table S1: Yearly GDP Per Capita** | | | |
| --- | --- | --- | --- |
| **Year** | **GDP (MYR)** | **Population** | **GDP Per Capita** |
| **2009** | 712,857,000,000 | 28,081,000 | 25,385.74 |
| **2010** | 821,400,000,000 | 28,589,000 | 28,731.33 |
| **2011** | 864,900,000,000 | 29,062,000 | 29,760.51 |
| **2012** | 912,300,000,000 | 29,510,000 | 30,914.94 |
| **2013** | 955,100,000,000 | 30,214,000 | 31,611.17 |
| **2014** | 1,012,400,000,000 | 30,709,000 | 32,967.53 |
| **2015** | 1,176,900,000,000 | 31,186,000 | 37,738.09 |
| **2016** | 1,229,300,000,000 | 31,634,000 | 38,860.09 |
| **2017** | 1,300,800,000,000 | 32,023,000 | 40,620.80 |
| **2018** | 1,363,800,000,000 | 32,382,000 | 42,115.99 |
| **2019** | 1,343,900,000,000 | 32,523,000 | 41,321.53 |

*Source: [1, 2] and author’s own calculation*

**References**

[1] DOSM. Malaysian Annual GDP - Department of Statistics Malaysia. 2009 - 2019. <https://newss.statistics.gov.my/newss-portalx/ep/epLogin.seam> (accessed on 2 June 2021)

[2] DOSM. Household Income Survey 2009 - 2019 Department of Statistics Malaysia. 2009 - 2019. <https://newss.statistics.gov.my/newss-portalx/ep/epProductFreeDownloadSearch.seam> (accessed on 12 June 2021)
