# Supplementary material for: Exploring the value in variations of the Relative Income Price (RIP) for calculating cigarette affordability: An illustration using Malaysia
Source: PLoS One. 2024 Nov 15;19(11):e0313695. doi: 10.1371/journal.pone.0313695 (PMC11567636; doi:10.1371/journal.pone.0313695)
Supplement: S2 Table — (DOCX) [file pone.0313695.s002.docx]

**Supporting Information to accompany “*Exploring the Value in Variations of the Relative Income Price (RIP) for Calculating Cigarette Affordability: An Illustration using Malaysia*”**

| **Table S2: Monthly Household Income Per Capita (HIPC) and Household Expenditure Per Capita (HEPC)** | | | | | | | | | | | | | |
| --- | --- | --- | --- | --- | --- | --- | --- | --- | --- | --- | --- | --- | --- |
| **Type** | **Category** | **Years** | **2009** | **2010*** | **2011*** | **2012** | **2013*** | **2014** | **2015*** | **2016*** | **2017*** | **2018*** | **2019** |
| **HIPC** | **Overall** | **Household Income (MYR)** | 4,025 |  |  | 5,000 |  | 6,141 |  | 6,958 |  |  | 7,901 |
|  |  | **Size of Household (person)** | 4.5 |  |  | 4.3 |  | 4.3 |  | 4.1 |  |  | 3.9 |
|  |  | **Household Income Per Capita (MYR)** | 894.44 | 983.89 | 1,073.34 | 1,162.79 | 1,295.46 | 1,428.14 | 1,562.61 | 1,697.07 | 1,806.68 | 1,916.29 | 2,025.90 |
|  | **Urban** | **Household Income (MYR)** | 4,705 |  |  | 5,742 |  | 6,833 |  | 7,671 |  |  | 8,635 |
|  |  | **Size of Household (person)** | 4.5 |  |  | 4.3 |  | 4.3 |  | 4.1 |  |  | 3.9 |
|  |  | **Household Income Per Capita (MYR)** | 1,045.56 | 1,135.01 | 1,224.46 | 1,335.35 | 1,468.02 | 1,589.07 | 1,723.54 | 1,870.98 | 1,980.59 | 2,090.20 | 2,214.10 |
|  | **Rural** | **Household Income (MYR)** | 2,545 |  |  | 3,080 |  | 3,831 |  | 4,359 |  |  | 5,004 |
|  |  | **Size of Household (person)** | 4.5 |  |  | 4.3 |  | 4.3 |  | 4.1 |  |  | 3.9 |
|  |  | **Household Income Per Capita (MYR)** | 565.56 | 655.01 | 744.46 | 716.28 | 848.95 | 890.93 | 1,025.40 | 1,063.17 | 1,172.78 | 1,282.39 | 1,283.08 |
| **HEPC** | **Overall** | **Household Expenditure (MYR)** |  |  |  |  |  | 3,412 |  | 4,033 |  |  | 4,534 |
|  |  | **Size of Household (person)** |  |  |  |  |  | 4.3 |  | 4.1 |  |  | 3.9 |
|  |  | **Household Expenditure Per Capita (MYR)** | 486.67 | 555.75 | 624.83 | 693.91 | 762.99 | 832.09 | 907.87 | 983.66 | 1,043.30 | 1,102.94 | 1,162.56 |
|  | **Urban** | **Household Expenditure (MYR)** |  |  |  |  |  | 3,921 |  | 4,402 |  |  | 4,916 |
|  |  | **Size of Household (person)** |  |  |  |  |  | 4.3 |  | 4.1 |  |  | 3.9 |
|  |  | **Household Expenditure Per Capita (MYR)** | 547.78 | 620.60 | 693.42 | 766.24 | 839.06 | 911.86 | 992.76 | 1073.66 | 1135.94 | 1198.22 | 1260.51 |
|  | **Rural** | **Household Expenditure (MYR)** |  |  |  |  |  | 2,027 |  | 2,284 |  |  | 2,443 |
|  |  | **Size of Household (person)** |  |  |  |  |  | 4.3 |  | 4.1 |  |  | 3.9 |
|  |  | **Household Expenditure Per Capita (MYR)** | 275.70 | 314.84 | 353.98 | 393.12 | 432.76 | 471.40 | 514.24 | 557.07 | 580.14 | 603.21 | 626.28 |

*Source: [1] and author’s own calculation*

**Years where estimation values apply.*

**References**

[1] DOSM. Household Income Survey 2009 - 2019 Department of Statistics Malaysia. 2009 - 2019. <https://newss.statistics.gov.my/newss-portalx/ep/epProductFreeDownloadSearch.seam> (accessed on 12 June 2021)
