# Supplementary material for: Exploring the value in variations of the Relative Income Price (RIP) for calculating cigarette affordability: An illustration using Malaysia
Source: PLoS One. 2024 Nov 15;19(11):e0313695. doi: 10.1371/journal.pone.0313695 (PMC11567636; doi:10.1371/journal.pone.0313695)
Supplement: S3 Table — (DOCX) [file pone.0313695.s003.docx]

**Supporting Information to accompany “*Exploring the Value in Variations of the Relative Income Price (RIP) for Calculating Cigarette Affordability: An Illustration using Malaysia*”**

| **Table S3: Consumption Estimates Per Person/Year for 2011 – 2019 in Rural Areas** | | | | | | | | | |
| --- | --- | --- | --- | --- | --- | --- | --- | --- | --- |
| **Band/Year** | **2011** | **2012*** | **2013*** | **2014*** | **2015*** | **2016*** | **2017*** | **2018*** | **2019** |
| **Ultra-Low Estimates** | 3,990.18 | 3,953.31 | 3,916.44 | 3,879.57 | 3,842.70 | 3,805.83 | 3,768.96 | 3,732.09 | 3,695.26 |
| **Lower Estimates** | 4,574.69 | 4,535.84 | 4,496.99 | 4,458.14 | 4,419.29 | 4,380.44 | 4,341.59 | 4,302.74 | 4,263.93 |
| **Central Estimates** | 5,082.99 | 5,039.83 | 4,996.67 | 4,953.51 | 4,910.35 | 4,867.19 | 4,824.03 | 4,780.87 | 4,737.70 |
| **Upper Estimates** | 5,591.29 | 5,543.81 | 5,496.33 | 5,448.85 | 5,401.37 | 5,353.89 | 5,306.41 | 5,258.93 | 5,211.47 |

**Years where linear interpolation values apply*

*Sources: [1] Authors own calculations*

**References**

[1] KKM. National Health and Morbidity Survey (NHMS). Kuala Lumpur: Institute for Public Health, Ministry of Health Malaysia 2010 - 2019.
