# Supplementary material for: Exploring the value in variations of the Relative Income Price (RIP) for calculating cigarette affordability: An illustration using Malaysia
Source: PLoS One. 2024 Nov 15;19(11):e0313695. doi: 10.1371/journal.pone.0313695 (PMC11567636; doi:10.1371/journal.pone.0313695)
Supplement: S4 Table — (DOCX) [file pone.0313695.s004.docx]

**Supporting Information to accompany “*Exploring the Value in Variations of the Relative Income Price (RIP) for Calculating Cigarette Affordability: An Illustration using Malaysia*”**

| **Table S4: Consumption Estimates Per Person/Year for 2011 – 2019 in Urban Areas** | | | | | | | | | |
| --- | --- | --- | --- | --- | --- | --- | --- | --- | --- |
| **Band/Year** | **2011** | **2012*** | **2013*** | **2014*** | **2015*** | **2016*** | **2017*** | **2018*** | **2019** |
| **Ultra-Low Estimates** | 4,148.96 | 4,058.39 | 3,967.82 | 3,877.25 | 3,786.68 | 3,696.11 | 3,605.54 | 3,514.97 | 3,424.43 |
| **Lower Estimates** | 4,750.27 | 4,655.33 | 4,560.39 | 4,465.45 | 4,370.51 | 4,275.57 | 4,180.63 | 4,085.69 | 3,990.78 |
| **Central Estimates** | 5,278.08 | 5,172.59 | 5,067.10 | 4,961.61 | 4,856.12 | 4,750.63 | 4,645.14 | 4,539.65 | 4,434.20 |
| **Upper Estimates** | 5,805.89 | 5,689.86 | 5,573.83 | 5,457.80 | 5,341.77 | 5,225.74 | 5,109.71 | 4,993.68 | 4,877.62 |

**Years where linear interpolation values apply*

*Source: [1] Authors own calculations*

**References**

[1] KKM. National Health and Morbidity Survey (NHMS). Kuala Lumpur: Institute for Public Health, Ministry of Health Malaysia 2010 - 2019.
