# Supplementary material for: Exploring the value in variations of the Relative Income Price (RIP) for calculating cigarette affordability: An illustration using Malaysia
Source: PLoS One. 2024 Nov 15;19(11):e0313695. doi: 10.1371/journal.pone.0313695 (PMC11567636; doi:10.1371/journal.pone.0313695)
Supplement: S5 Table — (DOCX) [file pone.0313695.s005.docx]

**Supporting Information to accompany “*Exploring the Value in Variations of the Relative Income Price (RIP) for Calculating Cigarette Affordability: An Illustration using Malaysia*”**

| **Table S5: Affordability Calculation Using Conventional Method (2000RIP)** | | | | | | | | | | | | | |
| --- | --- | --- | --- | --- | --- | --- | --- | --- | --- | --- | --- | --- | --- |
|  |  |  | 2009 | 2010 | 2011 | 2012 | 2013 | 2014 | 2015 | 2016 | 2017 | 2018 | 2019 |
| Gross Domestic Product (GDP) Per Capita | Overall |  | 3.1% | 3.0% | 2.9% | 2.9% | 3.3% | 3.6% | 4.1% | 4.0% | 3.8% | 3.8% | 3.9% |
| Household Income Per Capita (HIPC) | Overall |  | 7.3% | 6.7% |  |  |  | 7.0% |  | 7.6% |  |  | 6.6% |
|  | Urban |  | 6.2% | 5.8% |  |  |  | 6.3% |  | 6.9% |  |  | 6.0% |
|  | Rural |  | 11.5% | 10.8% |  |  |  | 11.2% |  | 12.1% |  |  | 10.4% |
| Household Expenses Per Capita (HEPC) | Overall |  |  |  |  |  |  | 12.0% |  | 13.1% |  |  | 11.5% |
|  | Urban |  |  |  |  |  |  | 11.0% |  | 12.0% |  |  | 10.6% |
|  | Rural |  |  |  |  |  |  | 17.7% |  | 19.4% |  |  | 17.1% |

*Source: Author’s own calculation*

*Note: Results are presented as percentages where the higher figures denote a higher proportion of the financial measures of wealth (GDP, HIPC and HEPC) required to purchase cigarettes. The higher the percentage, the less affordable tobacco to be.*
