# Supplementary material for: Exploring the value in variations of the Relative Income Price (RIP) for calculating cigarette affordability: An illustration using Malaysia
Source: PLoS One. 2024 Nov 15;19(11):e0313695. doi: 10.1371/journal.pone.0313695 (PMC11567636; doi:10.1371/journal.pone.0313695)
Supplement: S6 Table — (DOCX) [file pone.0313695.s006.docx]

**Supporting Information to accompany “*Exploring the Value in Variations of the Relative Income Price (RIP) for Calculating Cigarette Affordability: An Illustration using Malaysia*”**

| **Table S6: Affordability Calculation Using Actual Consumption Estimates (ConsumptionRIP) based on Overall Population** | | | | | | | | | | | | | |
| --- | --- | --- | --- | --- | --- | --- | --- | --- | --- | --- | --- | --- | --- |
|  |  |  | 2009 | 2010 | 2011 | 2012 | 2013 | 2014 | 2015 | 2016 | 2017 | 2018 | 2019 |
| GDP Per Capita | Overall | Central Estimates |  |  | 1.2% | 1.2% | 1.3% | 1.4% | 1.5% | 1.4% | 1.3% | 1.3% | 1.3% |
| HIPC | Overall |  |  |  |  |  |  | 2.7% |  | 2.7% |  |  | 2.2% |
|  | Urban |  |  |  |  |  |  | 2.5% |  | 2.5% |  |  | 1.9% |
|  | Rural |  |  |  |  |  |  | 4.1% |  | 4.3% |  |  | 3.9% |
| HEPC | Overall |  |  |  |  |  |  | 4.6% |  | 4.7% |  |  | 3.8% |
|  | Urban |  |  |  |  |  |  | 4.3% |  | 4.3% |  |  | 3.4% |
|  | Rural |  |  |  |  |  |  | 6.4% |  | 6.9% |  |  | 6.4% |

*Source: Author’s own calculation*

*Note: Results are presented as percentages where the higher figures denote a higher proportion of the financial measures of wealth (GDP, HIPC and HEPC) required to purchase cigarettes. The higher the percentage, the less affordable tobacco to be.*
