# Supplementary material for: Exploring the value in variations of the Relative Income Price (RIP) for calculating cigarette affordability: An illustration using Malaysia
Source: PLoS One. 2024 Nov 15;19(11):e0313695. doi: 10.1371/journal.pone.0313695 (PMC11567636; doi:10.1371/journal.pone.0313695)
Supplement: S8 Table — (DOCX) [file pone.0313695.s008.docx]

**Supporting Information to accompany “*Exploring the Value in Variations of the Relative Income Price (RIP) for Calculating Cigarette Affordability: An Illustration using Malaysia*”**

| **Table S8: Affordability Calculation Using Actual Consumption Estimates (ConsumptionRIP) based on Smokers Population** | | | | | | | | | | | | | |
| --- | --- | --- | --- | --- | --- | --- | --- | --- | --- | --- | --- | --- | --- |
|  |  |  | 2009 | 2010 | 2011 | 2012 | 2013 | 2014 | 2015 | 2016 | 2017 | 2018 | 2019 |
| GDP Per Capita | Overall | Ultra-Low |  |  | 5.9% | 5.9% | 6.6% | 7.1% | 7.8% | 7.4% | 7.0% | 6.8% | 6.8% |
|  |  | Lower |  |  | 6.7% | 6.7% | 7.5% | 8.1% | 9.0% | 8.6% | 8.1% | 7.9% | 7.9% |
|  |  | Central |  |  | 6.8% | 7.1% | 6.8% | 6.8% | 6.8% | 9.5% | 8.9% | 8.5% | 8.5% |
|  |  | Upper |  |  | 8.2% | 8.2% | 9.2% | 9.9% | 11.0% | 10.5% | 9.8% | 9.6% | 9.6% |
| Household Income Per Capita (HIPC) | Overall | Ultra-Low |  |  |  |  |  | 13.6% |  | 14.2% |  |  | 11.5% |
|  |  | Lower |  |  |  |  |  | 15.6% |  | 16.4% |  |  | 13.4% |
|  |  | Central |  |  |  |  |  | 17.3% |  | 18.2% |  |  | 14.8% |
|  |  | Upper |  |  |  |  |  | 19.1% |  | 20.0% |  |  | 16.3% |
|  | Urban | Ultra-Low |  |  |  |  |  | 12.2% |  | 12.8% |  |  | 10.3% |
|  |  | Lower |  |  |  |  |  | 14.1% |  | 14.8% |  |  | 12.0% |
|  |  | Central |  |  |  |  |  | 15.6% |  | 16.4% |  |  | 13.4% |
|  |  | Upper |  |  |  |  |  | 17.2% |  | 18.0% |  |  | 14.7% |
|  | Rural | Ultra-Low |  |  |  |  |  | 21.8% |  | 23.1% |  |  | 19.2% |
|  |  | Lower |  |  |  |  |  | 25.0% |  | 26.6% |  |  | 22.2% |
|  |  | Central |  |  |  |  |  | 27.8% |  | 29.6% |  |  | 24.6% |
|  |  | Upper |  |  |  |  |  | 30.6% |  | 32.5% |  |  | 27.1% |
| Household Expenses Per Capita (HEPC) | Overall | Ultra-Low |  |  |  |  |  | 23.3% |  | 24.4% |  |  | 20.0% |
|  |  | Lower |  |  |  |  |  | 26.8% |  | 28.2% |  |  | 23.3% |
|  |  | Central |  |  |  |  |  | 29.8% |  | 31.4% |  |  | 25.9% |
|  |  | Upper |  |  |  |  |  | 32.8% |  | 34.5% |  |  | 28.5% |
|  | Urban | Ultra-Low |  |  |  |  |  | 21.3% |  | 22.2% |  |  | 18.1% |
|  |  | Lower |  |  |  |  |  | 24.5% |  | 25.7% |  |  | 21.1% |
|  |  | Central |  |  |  |  |  | 27.2% |  | 28.6% |  |  | 23.5% |
|  |  | Upper |  |  |  |  |  | 29.9% |  | 31.4% |  |  | 25.8% |
|  | Rural | Ultra-Low |  |  |  |  |  | 34.3% |  | 37.0% |  |  | 31.6% |
|  |  | Lower |  |  |  |  |  | 39.4% |  | 42.6% |  |  | 36.5% |
|  |  | Central |  |  |  |  |  | 43.8% |  | 47.3% |  |  | 40.5% |
|  |  | Upper |  |  |  |  |  | 48.2% |  | 52.0% |  |  | 44.6% |

*Source: Author’s own calculation*

*Note: Results are presented as percentages where the higher figures denote a higher proportion of the financial measures of wealth (GDP, HIPC and HEPC) required to purchase cigarettes. The higher the percentage, the less affordable tobacco to be.*
