# Supplementary material for: Exploring the value in variations of the Relative Income Price (RIP) for calculating cigarette affordability: An illustration using Malaysia
Source: PLoS One. 2024 Nov 15;19(11):e0313695. doi: 10.1371/journal.pone.0313695 (PMC11567636; doi:10.1371/journal.pone.0313695)
Supplement: Attachment — Submitted filename: Review.docx [file pone.0313695.s010.docx]

First, I want to thank the authors and the editor for the opportunity to review this paper. The study reports a detailed timeline of cigarette affordability using diverse methods to measure affordability. Recommending using household/income expenditure per capita instead of GDP measures allows for analysis of the change in affordability across different demographic groups which would be useful to improve tobacco control policies. However, the measures using yearly consumption of cigarettes are not comparable across years as endogeneity exists between price and consumption. I would recommend addressing this issue before being ready for publication.

My specific comments are shown below.

Comment 1: If consumption changes between years, then the estimates are not comparable across years. A decrease in ConsumptionRIP can be caused by (i) a decrease in consumption, (ii) a price decrease, and (iii) an increase in household income. The relationship between consumption and price leads to an endogeneity problem, as increases in price during the year $i$ would decrease consumption (by the price elasticity), which the authors are aware of. With 2,000 cigarettes (or any other constant quantity), a price increase would reduce affordability:

$${2000RIP\_HEPC}_{i}=\frac{2000\cdot\boldsymbol{\uparrow}{Price}_{i}}{{HEPC}_{i}}$$

However, in the ConsumptionRIP measures, the measure would change something like this:

$${ConspumptionRIP\_HEPC}_{i}=\frac{\boldsymbol{\downarrow}Cons\cdot\boldsymbol{\uparrow}{Price}_{i}}{{HEPC}_{i}}$$

If the ConsumptionRIP increases or decreases then will be determined by the price elasticity; if it’s inelastic, the consumption would decrease less than the price increase, so it would become less affordable, but if it's elastic then it would be more affordable.

Because of these interpretation issues, I don’t recommend using ConsumptionRIP for the analysis of affordability. What it would be useful to interpret is to define a year to use as a baseline. For example, set at the Consumption level of 2009. That way the interpretation would be if it’s less or more affordable to buy the average consumption of 2009.

Comment 2: Does the law require to adjust the specific tax by the inflation every X years? Does the increase happen automatically or a new law is required to adjust taxes by inflation? An effective way to ensure adjusting by inflation is to include it in the tobacco law.

Comment 3: In the discussion section, it is mentioned that the differences are significant. Was a test used to assess the significance? It should be stated which test and significance level. If no test was used, I recommend changing the wording.

Comment 4: The use of household expenditure and income is a great idea to measure how affordability changes across income groups. In the introduction was mentioned that one of the advantages of HEPC/HIPC measures is the analysis by income groups, but this was not done (apart from urban and rural was expecting that type of analysis and can be an important contribution that doesn’t require much more work than doing the same analysis as urban and rural, but, for example, by deciles of HEPC/HIPC. Has the changes in affordability been different for lower and higher-income groups?
