# Supplementary material for: Exploring the value in variations of the Relative Income Price (RIP) for calculating cigarette affordability: An illustration using Malaysia
Source: PLoS One. 2024 Nov 15;19(11):e0313695. doi: 10.1371/journal.pone.0313695 (PMC11567636; doi:10.1371/journal.pone.0313695)
Supplement: Attachment — Submitted filename: Paper 2_Response to Reviewers_V3.1.docx [file pone.0313695.s011.docx]

Response to Editor’s and Reviewers’ Comments

**Exploring the Value in Variations of the Relative Income Price (RIP) for Calculating Cigarette Affordability: An Illustration using Malaysia**

**We thank the editor and reviewers for their kind comments and for the very helpful suggestions they made for how we can improve the paper. It was gratifying that all reviewers found value in the paper and were generally very supportive of it. We have made some revisions to the paper in light of the helpful comments received, and provide further details of our response to each comment received in the paragraphs below in red.**

Editor’s comment:

1) Please ensure that your manuscript meets PLOS ONE's style requirements, including those for file naming. The PLOS ONE style templates can be found at:

https://journals.plos.org/plosone/s/file?id=wjVg/PLOSOne_formatting_sample_main_body.pdf and

https://journals.plos.org/plosone/s/file?id=ba62/PLOSOne_formatting_sample_title_authors_affiliations.pdf.

**Thank you for the reminder to follow PLOS ONE’s style requirements. We have ensured that our manuscript meets these requirements, including proper file naming.**

2. Thank you for stating the following in the Competing Interests section:

[JRB owns 10 shares in Imperial Brands for research purposes. The shares were a gift from a public health campaigner and are not held for financial gain or benefit. All dividends received are donated to health related charities, and proceeds from any future share sale or takeover will be similarly donated.].

Please confirm that this does not alter your adherence to all PLOS ONE policies on sharing data and materials, by including the following statement: ""This does not alter our adherence to PLOS ONE policies on sharing data and materials.” (as detailed online in our guide for authors http://journals.plos.org/plosone/s/competing-interests). If there are restrictions on sharing of data and/or materials, please state these. Please note that we cannot proceed with consideration of your article until this information has been declared.

Please include your updated Competing Interests statement in your cover letter; we will change the online submission form on your behalf.

**Thank you for your instructions regarding the Competing Interests section. There are no restrictions on the sharing of data and/or materials. Therefore we have updated our Competing Interests statement accordingly and included it in our cover letter as below:**

**Regarding my competing interest statement we can confirm that “This does not alter our adherence to PLOS ONE policies on sharing data and materials” and that we are happy for you to update our online submission form in this regard.**

3. We note that your Data Availability Statement is currently as follows: [All data used herein is publicly available and obtained from the sources listed within the manuscript.]

Please confirm at this time whether or not your submission contains all raw data required to replicate the results of your study. Authors must share the “minimal data set” for their submission. PLOS defines the minimal data set to consist of the data required to replicate all study findings reported in the article, as well as related metadata and methods (https://journals.plos.org/plosone/s/data-availability#loc-minimal-data-set-definition).

For example, authors should submit the following data:

- The values behind the means, standard deviations and other measures reported;

- The values used to build graphs;

- The points extracted from images for analysis.

Authors do not need to submit their entire data set if only a portion of the data was used in the reported study.

If your submission does not contain these data, please either upload them as Supporting Information files or deposit them to a stable, public repository and provide us with the relevant URLs, DOIs, or accession numbers. For a list of recommended repositories, please see https://journals.plos.org/plosone/s/recommended-repositories.

If there are ethical or legal restrictions on sharing a de-identified data set, please explain them in detail (e.g., data contain potentially sensitive information, data are owned by a third-party organization, etc.) and who has imposed them (e.g., an ethics committee). Please also provide contact information for a data access committee, ethics committee, or other institutional body to which data requests may be sent. If data are owned by a third party, please indicate how others may request data access.

**Thank you very much for your queries regarding data availability. We confirm that our submission contains all raw data required to replicate the results of our study, which are presented as the "minimal data set" in the Supporting Information as “File S9: Raw Data used for Study Results.” This file contains individual tabs representing the tables and figures used in the study.**

**Specifically, we have submitted the following data:**

- **The values behind the measures reported (means, standard deviations, etc.)**
- **The values used to build graphs**
- **The points extracted from images for analysis**

**Additionally, we have provided the supporting data as Supporting Information (Table S1 to Table S8) files.**

**We also confirm no ethical or legal restrictions on sharing a de-identified data set. All data used herein were produced by Malaysian government entities and are publicly available.**

**Reviewer 1’s comments:**

The authors propose a method to measure cigarette affordability, to improve the classical relative income price (RIP) based on GDP per capita (Blecher & van Walbeek, 2004). The method is based on household income per capita (HIPC) and household expenditure per capita (HEPC). The new measure suggests that a stronger tax increase is needed for Malaysia than the calculations based on the GDP-RIP.

As mentioned in the document, the idea was used before for China (16) and Russia (8), in order to allow for a measure that captures heterogeneity across the income distribution. They base their analysis on the disposable household income per capita; several categories are made in or. Their measures kept the 20cigs x 100 packs, for specific sets of brands, as the standard baskets of consumption. The authors contributions are (i) to consider expenditures rather than income, (ii) to define a ‘moving’ basket (central consumption estimates).

**We are glad to see that our writing was able to convey clearly the key points we wanted to explore in the paper. We are sincerely grateful for your thoughtful feedback and comments, which we address individually in the paragraphs below.**

1) In my opinion, the RIP was designed for comparability across countries as a simple measure that provides a rapid answer to the question: are our cigarettes expensive or not? By adding the heterogeneity extension, is the possible to state if the cigarettes are expensive or not for a given set of the population. The concept behind this paper is a bit more complex as it aims to be micro-founded on the decisions to quit or change consumption. In other words, it is linked to elasticities per se, then it is subject to all the endogeneity considerations of this statistical object: there is selection on who smoke, and what do they smoke, and how frequent. Those are the implications of deciding to allow for a basket that is dependent on the information of the survey; even if it is used as an aggregate. Such limitations should be acknowledged in the discussion section.

**We agree with you that the Relative Income Price (RIP) was designed to provide a straightforward metric as it compares the cost of 2,000 cigarette sticks as a proportion of GDP, allowing for temporal and international comparisons. This is something that is very valuable but also inherently limited, so, we have introduced additional complexities to this framework in order to develop further insights.**

**As you note one implication of the complexities we have introduced by considering actual consumption levels, is the issue of endogeneity. We did this because we felt it was helpful to consider the typical cost of being a person who smokes. As such we are looking to explore both the affordability of tobacco (the RIP using the fixed 2,000 sticks) but also the affordability of being a typical smoker at different points in time (the RIP with the actual consumption estimates).**

**Endogeneity is a common concern, particularly in econometric regression analyses, because it can lead to biased estimations. In such cases, the coefficients derived may be inaccurate and not reflect true relationships within the data (see for example, https://www.sciencedirect.com/topics/psychology/endogeneity). Since our study does not involve regression analysis, this concern does not apply.**

**What our approach does mean is that the versions of the RIP featuring actual consumption estimates are in practice exploring the overall impact of consumer behaviour, prices, and incomes/wealth, whereas the traditional RIP only explores prices and incomes. The endogeneity concern is therefore that consumer behaviour is in part determined by prices. With higher prices, the well established inelastic nature of tobacco products means that consumers will respond to some extent with slightly lower levels of consumption. As such what our traditional 2,000 stick RIP and our consumption RIP are allowing is an exploration of the impact of this consumer behaviour, as that is the cause of the difference between the different versions of the RIP. We think this is valuable in a market like Malaysia where there are different consumption levels in different parts of the economy, and where levels of illicit are high. This mean that many consumers might not be exposed to the price of legal cigarettes, or might be exposed to a different extent, and such detail wouldn’t be possible to explore with a single elasticity value (please also see our response below to your next point about the use of elasticity estimates).**

**Our study is therefore looking to offer a range of insights into the issue of affordability with the range of RIP series we provide, rather than rely solely on a single metric. Omitting the complexities associated with the versions of the RIP would simplify the analysis but at the cost of losing important details that contribute to a thorough understanding of tobacco affordability dynamics.**

**To outline this rationale, we have adjusted the methodology section of the revised paper (see p.10) where we now incorporate a new section entitled “*Endogeneity between price and consumption levels*”. This section outlines the potential endogeneity between price and consumption levels when examining the relationship between cigarette consumption and price. The section also explains the use of different RIP series in response to price changes in Malaysia, considering the substantial presence of an illicit tobacco market.**

**We understand your suggestion to include this point in the limitations section. However, we chose to incorporate it into the methods section so that we can capture the complexity of the issue early on. By detailing it in the methods, we aimed to provide a more comprehensive understanding of the approach and its underlying intricacies, ensuring that the nuances are appropriately conveyed to the readers. We also note that the discussion section already reflects back on the endogeneity issue (p.22) so it should be something the reader is now fully able to consider holistically from the main body of the paper.**

2) The authors might consider keeping the basket fixed and define an additional concept that is linked to the peer-reviewed consumption elasticities estimates for the country. In this way, it is easier to contribute to the discussion without the hazard of using constructs that are a direct function of the price.

**Thank you for your suggestion. However, the country's most recent and only elasticity estimates were produced for the year 2009 and were published in 2013 (See ‘*An Optimal Cigarette Tax in Malaysia’* by MN, Norashidah et al.** [**http://www.ijem.upm.edu.my/vol7no2/bab02.pdf**](http://www.ijem.upm.edu.my/vol7no2/bab02.pdf)**). As such this data is outdated, as the market has seen a consider increase in the illicit market during this time, with our own work showing it has gone from 40.7% in 2011 to 69.9% in 2019 – see** [**https://doi:10.1136/tc-2023-058333**](https://doi:10.1136/tc-2023-058333)**.**

**As noted above in our response to your first comment, we believe there is value in presenting a range of different version of the RIP including those with both fixed consumption volumes, and those based on actual consumption estimates. In order to better explain the value of this approach we have made the various changes as outlined in that answer, most notably in the new section on endogeneity in the methodology section.**

**We hope this will help readers understand why we present such a variety of data series and see the value in them. However, we note that including versions that allow consumption to changes doesn’t in any way impact the versions of the RIP with the fixed 2000 stick consumption level. As such any reader who remains concerned by the endogeneity issue will be able to concentrate on the traditional RIP measures if they so wish.**

3) An important issue is the lack of standard errors. The HIPC and HEPC estimates are based on survey data. The authors can perform a simple ‘box’ exercise for the 2000RIP estimates (i.e., use the lower and upper bound of the CI for income), but for the ConsumptionRIP a bootrapping strategy is required as both the numerator and the denominator are constructed with survey data. This is central to be able to say that there are actual differences between the strategies considered.

**Thank you for your valuable feedback. Unfortunately, the government reports from which the HIPC, HEPC, and consumption data was sourced presented only point estimates. The report from which HIPC and HEPC was sourced does have an accompanying technical note that acknowledges the potential for sampling and non-sampling errors. However, it did not provide confidence intervals (except for one illustrative example) to account for these errors. The technical note accompanying the consumption data adopts the same approach. Therefore, given the lack of confidence intervals and associated information, we were unable to conduct a wider statistical analysis in the manner you have suggested.**

**To account for this inherent weakness in the survey derived data utilised, we have expanded the limitations section to cover the point. The paragraph covering survey limitations now (p.26) reads as:**

*“The study is subject to several limitations. Firstly, HIPC and HEPC were produced from survey data which may lead to predisposition, as the surveys do not cover residential institutions (e.g., hotels, hostels, hospitals, and senior citizens’ home) which might slightly bias the data and hence the results herein. They were also not available on a yearly basis preventing analysis in all years.* *Furthermore, the surveys used to generate estimates of HIPC, HEPC, and consumption levels were presented as point estimates only. No confidence intervals were provided (even in the accompanying technical reports) and as such, wider statistical analysis could not be performed.”*

4) Minor comments

HEPC acronym should be included at the same time as the HIPC

**We appreciate your thorough review and attention to detail. We apologise that we did not include the HEPC acronym in the abstract section where it was first introduced, and we have now corrected this error. We have also carefully checked the rest of the manuscript with aim of ensuring that all acronyms are consistently identified on first use throughout the manuscript.**

**Reviewer 2’s comments:**

I think the paper is interesting. The discussion of tobacco taxation and health impacts is quite relevant. However, I have some concerns about methodological choices to obtain the affordability measures.

**We are glad that you find this paper interesting. In the paragraphs below we address the specific queries raised.**

1) Why choose a fixed annual consumption of 2,000 sticks? What is the rationale on this? Is it the same for urban and rural areas? If you have a survey on that you should used and be clear on this. I am quite confused on how this went on.

**Thank you for your comment regarding the selection of the fixed annual consumption of 2,000 sticks. This choice is simply our adopting the conventional RIP calculation, which utilises 2,000 sticks (as well as GDP data), and which was first proposed by Blecher and van Walbeek (2004) and which has subsequently been used many other analyses since then – with 278 times in Google Scholar. The idea is that it applies to the economy as a whole, and hence in our context, covers those in both urban and rural areas.**

**The 2,000 sticks were chosen by Blecher and van Walbeek because it is a round number that approximates the annual consumption of an average smoker, making it a relevant and practical benchmark for measuring affordability over a year.**

**In our study, the only time we move away from using 2,000 sticks is when we use estimated consumption values and these are identified as being the ConsuptionRIP.**

**We have revised the manuscript to clearly explain the use of RIP, with a particular focus on the level of consumption assumed. In particular, we have changed the following section:**

**Introduction (Page 3): *“… and the most widely used, the Relative Income Price (RIP), which is the proportion of a nation’s annual Gross Domestic Product (GDP) per capita needed to purchase 2,000 cigarette sticks (adopted as a round number approximation of typical per capita consumption levels)”***

**Clarification that the same measures are used in the rural and urban areas is offered in the methodology (Page 7), which reads as:**

***“Recognising the limitation of the conventional RIP calculation which presumes a fixed annual cigarette consumption of 2,000 sticks and may not represent the true habits of people who smoke in Malaysia, we then integrated yearly cigarette consumption estimates (measured in sticks) into our calculations. We explored these measures of consumption in both rural and urban areas.”***

2) I do not think the measure you are using is indeed an affordability measure. If we think in household level it is ok. But, in a agreggated form I do not think so. It looks like a productivity difference between the tobacco industry and the average income worker (using gdp per capita, income or expenditure). If this is true, the affordability is expected to drop for most countries, since the tobacco industry is more productive than the average worker sector for most countries. This is my main concern.

**As noted in our response to your first point, the RIP measure of tobacco affordability is widely recognised (and used) in the tobacco control literature. Blecher and van Walbeek (2004) pioneered the concept of Relative Income Price (RIP) with the expressed purpose of assessing cigarette affordability, both over time and between countries. It has since been adopted by the authors of other studies, including studies from the World Health Organization (WHO) and the World Bank.**

**The RIP is calculated as the proportion of a nation’s annual Gross Domestic Product (GDP) per capita needed to purchase 2,000 cigarette sticks. As such it offers a summary of the situation as experienced by a typical individual in the country – the amount of income such a typical individual would have in the year and then the proportion of that required to buy 2,000 sticks (which is an approximation of typical per capita consumption volumes). Tobacco therefore becomes less affordable when prices increase, and becomes more affordable when incomes increase.**

**Tobacco prices are primarily driven by tobacco taxation rather than other factors such as industry productivity. See for example, the World Health Organization on the importance of taxation to tobacco prices:** [**https://www.who.int/data/gho/data/themes/topics/raise-taxes-on-tobacco**](https://www.who.int/data/gho/data/themes/topics/raise-taxes-on-tobacco)**.**

**In our study we utilise various version of the RIP but these ultimately these all look to explore how much of a typical individual’s wealth is required to buy cigarettes. In order to clarify the goals and approach of the paper, we have made various changes in the introduction (p.3 to p.5) and methodology (p.10) sections in light of this comment and the other comments received from the reviewers.**

**Reviewer 3’s comments:**

First, I want to thank the authors and the editor for the opportunity to review this paper. The study reports a detailed timeline of cigarette affordability using diverse methods to measure affordability. Recommending using household/income expenditure per capita instead of GDP measures allows for analysis of the change in affordability across different demographic groups which would be useful to improve tobacco control policies. However, the measures using yearly consumption of cigarettes are not comparable across years as endogeneity exists between price and consumption. I would recommend addressing this issue before being ready for publication.

**Thank you for taking the time to review our paper and for your valuable feedback. We appreciate your thoughtful comments and suggestions, which we believe will greatly enhance the quality and clarity of our study. We are pleased that you found value in our use of diverse methods to measure affordability. We agree that understanding the nuances of affordability across different demographic groups is crucial for informing effective tobacco control policies. We will address your queries regarding the use of yearly consumption of cigarettes and endogeneity below.**

Comment 1: If consumption changes between years, then the estimates are not comparable across years. A decrease in ConsumptionRIP can be caused by (i) a decrease in consumption, (ii) a price decrease, and (iii) an increase in household income. The relationship between consumption and price leads to an endogeneity problem, as increases in price during the year i would decrease consumption (by the price elasticity), which the authors are aware of. With 2,000 cigarettes (or any other constant quantity), a price increase would reduce affordability:

$$,2000RIP\_HEPC-i.=,2000\cdot\boldsymbol{\uparrow},Price-i.-,HEPC-i..$$

However, in the ConsumptionRIP measures, the measure would change something like this:

$$,ConspumptionRIP\_HEPC-i.=,\boldsymbol{\downarrow}Cons\cdot\boldsymbol{\uparrow},Price-i.-,HEPC-i..$$

If the ConsumptionRIP increases or decreases then will be determined by the price elasticity; if it’s inelastic, the consumption would decrease less than the price increase, so it would become less affordable, but if it's elastic then it would be more affordable.

Because of these interpretation issues, I don’t recommend using ConsumptionRIP for the analysis of affordability. What it would be useful to interpret is to define a year to use as a baseline. For example, set at the Consumption level of 2009. That way the interpretation would be if it’s less or more affordable to buy the average consumption of 2009.

**As you note one implication of the complexities we have introduced by considering actual consumption levels, is the issue of endogeneity. We did this because we felt it was helpful to consider the typical cost of being a person who smokes. As such we are looking to explore both the affordability of tobacco (the RIP using the fixed 2,000 sticks) but also the affordability of being a typical smoker at different points in time (the RIP with the actual consumption estimates).**

**Endogeneity is a common concern, particularly in econometric regression analyses, because it can lead to biased estimations. In such cases, the coefficients derived may be inaccurate and not reflect true relationships within the data (see for example, https://www.sciencedirect.com/topics/psychology/endogeneity). Since our study does not involve regression analysis, this concern does not apply.**

**What our approach does mean is that the versions of the RIP featuring actual consumption estimates are in practice exploring the overall impact of consumer behaviour, prices, and incomes/wealth, whereas the traditional RIP only explores prices and incomes. The endogeneity concern is therefore that consumer behaviour is in part determined by prices. With higher prices, the well established inelastic nature of tobacco products means that consumers will respond to some extent with slightly lower levels of consumption. As such what our traditional 2,000 stick RIP and our consumption RIP are allowing is an exploration of the impact of this consumer behaviour, as that is the cause of the difference between the different versions of the RIP. We think this is valuable in a market like Malaysia where there are different consumption levels in different parts of the economy and where levels of illicit are high. This mean that many consumers might not be exposed to the price of legal cigarettes, or might be exposed to a different extent, which can only be exploring by considering consumption values.).**

**We could, as you have suggested, have taken the estimated consumption level of a particular year (say 2009) and then simply looked to use that in all years. That would have made the traditional RIP more reflective of consumer experiences but we felt doing so wouldn’t offer any great additional insight over the traditional 2,000 stick RIP. All it would do is change the level at which the RIP fluctuates. For example, if we took 3,000 sticks then the proportion of income required would be 50% higher than the 2,000 stick RIP for all years. The pattern of changes would be exactly the same, just at a higher proportion of income/wealth required.**

**By allowing consumption to change each year we are looking to offer a range of different insights into the issue of affordability by virtue of the range of RIP series we provide. However, we note that including a version which allows for changes to consumption doesn’t in any way impact the versions of the RIP with the fixed 2000 stick consumption level. As such any reader who remains concerned by the endogeneity issue will be able to concentrate on the traditional RIP measures if they so wish. Omitting the complexities associated with the versions of the RIP that allow consumption to change would, we feel, simplify the paper but at the cost of losing important details that contribute to a thorough understanding of tobacco affordability dynamics.**

**In light of this comment and similar comments from another referee, we have revised the paper in several places. In particular we have adjusted the methodology section (see p.10) where we now incorporate a new section entitled “*Endogeneity between price and consumption levels”.* This section outlines the potential endogeneity between price and consumption levels when examining the relationship between cigarette consumption and price. The section also explains the use of different RIP series in response to price changes in Malaysia, considering the substantial presence of an illicit tobacco market. We also note that we return to the issue of the endogeneity in the discussion section (p.22).**

Comment 2: Does the law require to adjust the specific tax by the inflation every X years? Does the increase happen automatically or a new law is required to adjust taxes by inflation? An effective way to ensure adjusting by inflation is to include it in the tobacco law.

**The tobacco tax system in Malaysia does not feature an automatic adjustment for inflation every year, or indeed, in any year.** **Tobacco taxation adjustments require a new law each time they are changed, meaning that each change, even one just to account for inflation, requires a clear policy choice by the government.**

**To clarify this point we have revised the introduction (p.6) where we introduce the extent of tax increases in Malaysia since 2010. The relevant text now reads as:**

***“However, since 2010, tobacco tax increases in Malaysia have been infrequent as there are no routine adjustments to account for the impact of inflation”***

**Furthermore, we have also adjusted the discussion section to remind the reader of this important point when we discuss our results. The amended text (P. 25) now reads as:**

**“*Implementing effective tobacco tax policies in Malaysia requires a comprehensive approach that addresses both legislative and enforcement challenges. The increase in taxes in Malaysia does* *not entail an adjustment for inflation every year, or indeed, in any year. Tobacco taxation adjustments require a new law annually to adjust the taxes, meaning that each change, even one just to account for inflation, requires a clear policy choice. This legislative requirement highlights the importance of deliberate policy-making to ensure that tax changes are both effective and sustainable.*”**

Comment 3: In the discussion section, it is mentioned that the differences are significant. Was a test used to assess the significance? It should be stated which test and significance level. If no test was used, I recommend changing the wording.

**Thank you for highlighting this poor choice of wording, which we have now changed, as no statistical test was conducted. The amended text (p.16) now reads as:**

**“*However, it then decreased to 40.5% in 2019, representing a substantial difference of 6.8 percentage points with -14.38% affordability change*”**

Comment 4: The use of household expenditure and income is a great idea to measure how affordability changes across income groups. In the introduction was mentioned that one of the advantages of HEPC/HIPC measures is the analysis by income groups, but this was not done (apart from urban and rural was expecting that type of analysis and can be an important contribution that doesn’t require much more work than doing the same analysis as urban and rural, but, for example, by deciles of HEPC/HIPC. Has the changes in affordability been different for lower and higher-income groups?

**We are glad that you like the concept of using HEPC and HIPC, and appreciate the suggestion of using the deciles of HEPC/HIPC instead of utilising the difference in income between rural and urban areas.**

**However, measurement of financial series using deciles was unavailable until 2016, when the Malaysian government introduced a new income classification system. This system, based on the DOSM Household Income and Basic Amenities (HIS/BA) survey, divides the population into three distinct groups: Top 20% (T20), Middle 40% (M40), and Bottom 40% (B40). It wasn't until 2022 that the classification of income/expenditure on cigarettes was included in this system, which is why it was not used in the paper.**

**We agree that this classification would possibly provide clearer understanding of the income/expenditure associated with cigarette consumption, which, in turn, allows for a more nuanced approach when examining the impact of cigarette affordability on different segments of society and socio-economic strata. However, it simply is not possible to for the years we are looking to analyse.**

**As such, the rural and urban measures were chosen for our study to compare income levels, as they are the only split available. Rural areas represent the low-income group, while urban areas represent the high-income group. In order to clarify this point in the paper we have revised the introduction section where we first introduced the rural and urban areas (p.5), in order to explicitly mention the difference between these income groups. The revised text now reads as:**

***“To achieve this, we also employ a unique approach that incorporates actual cigarette consumption patterns, thereby taking into consideration the distinctions that exist between rural and urban areas. This distinction between areas is valuable as the data shows that rural areas are relatively low-income groups while urban areas are the relatively high-income groups. This method not only enhances our understanding of cigarette affordability but also allows us to explore the impact of consumer behaviour from different incomes groups in mitigating adverse changes in affordability.”***

**We have also revised the data section to clarify that the urban and rural split represents the best data available by adding in the text (p.9): “*The use of the urban and rural split to explore different consumer incomes/wealth levels was utilised as such information by decile or other such breakdown of levels was not available for the years being considered.”***
