# Supplementary material for: Exploring the value in variations of the Relative Income Price (RIP) for calculating cigarette affordability: An illustration using Malaysia
Source: PLoS One. 2024 Nov 15;19(11):e0313695. doi: 10.1371/journal.pone.0313695 (PMC11567636; doi:10.1371/journal.pone.0313695)
Supplement: Attachment — Submitted filename: Paper 2_Response to Reviewers_V4.docx [file pone.0313695.s012.docx]

Response to Editor’s and Reviewers’ Comments

**Exploring the Value in Variations of the Relative Income Price (RIP) for Calculating Cigarette Affordability: An Illustration using Malaysia**

**We thank the editor and reviewers for their help and support in improving the paper. We are particularly pleased that all reviewers found merit in the study and were generally supportive of its contributions in the revised paper, with just two remaining referee and editorial queries to address. We therefore provide a detailed response to address each of these two queries in the paragraphs below in red.**

Journal Requirements:

Please review your reference list to ensure that it is complete and correct. If you have cited papers that have been retracted, please include the rationale for doing so in the manuscript text, or remove these references and replace them with relevant current references. Any changes to the reference list should be mentioned in the rebuttal letter that accompanies your revised manuscript. If you need to cite a retracted article, indicate the article’s retracted status in the References list and also include a citation and full reference for the retraction notice.

**We have carefully reviewed the reference list to ensure its accuracy and completeness. We confirm that no retracted papers have been cited or used in the manuscript. As such, no changes to the reference list are necessary.**

Reviewer #3 comment:

Thanks for your response to my comments and for addressing all of them.

I have no major comments regarding my previous comments. I still think that consumption measures can be misinterpreted without a closer look at the change in average consumption and household expenditure and income. However, the authors provide this information (in the paper tables and supplemental material. Moreover, in the discussion section, the authors interpret the consumption measures in light of the changes in consumption and income.

**We are glad the reviewer feels the information we have included in the revised paper and its supplementary materials is sufficient to address their previous comments. Again, we thank the reviewer for their constructive input.**

I recommended a minor revision only because of one issue. If I understand correctly, the consumption HIPC and HEPC measures can be interpreted as the (annual) budget share of households dedicated to tobacco. If I am correct, these budget shares seem too large, suggesting that, in 2019, 25.9% of all household expenses (HEPC) were dedicated to tobacco products (and 40.5% in rural households). It is possible that these numbers are correct and that (smokers) rural households devote 40.5% of their expenses to tobacco, but these would be a lot higher than what the literature has found. A paper titled "Distribution of Household Tobacco Expenditure and Household Affordability of Tobacco Products in Malaysia" measured the budget share of tobacco products in Malaysia using the Household Expenditure Surveys and found that the overall budget share for tobacco products was 4.6%. Looking at other countries' estimates, the budget shares are closer to 5% than 26%.

These differences should be addressed in the paper, as indicating that the average rural smoker devotes 40.5% of their expenses to tobacco products is a "big" statement.

**Thank you for your thoughtful feedback and for raising the concern regarding the size of the Household Expenditure per Capita (HEPC) and Household Income per Capita (HIPC) measures.**

**In order to be sure of our results we have carefully reviewed our data and calculations, and while we acknowledge that our income/expenditure estimates seem a bit high in comparison to existing literature, we can confirm that the values are correct given our dataset and methodological framework adopted. We therefore think these figures reflect the reality of rural smoker households, which may face substantial socio-economic pressures as a result. This could result in a higher income allocation to tobacco products than other needs which could be an important finding for politicians.**

**We appreciate your reference to the paper, *"Distribution of Household Tobacco Expenditure and Household Affordability of Tobacco Products in Malaysia"*, which reports a national average tobacco budget share of 4.6%. Upon reviewing this paper, we note that the suggested figure isn’t directly comparable to the estimates in our paper. The 4.6% expenditure level is for the entire population of households whereas our study looks at the cost for individuals who use tobacco. Furthermore, in the aforementioned paper it says that the proportion of households reporting tobacco expenditure has never dropped below 35% which means that most households covered do not in fact spend any money on tobacco. As such a rough estimate would be that the expenditure in only those households who report tobacco consumption would be approximately three times the reported level or around 15%. While this is still lower than our own estimate of (for example) 26% for overall household expenses in 2019, it is far closer than the numbers might initially suggest. Furthermore, we report in our paper (p.16) that in the appendix we include more directly comparable estimates. For example, our expenditure based affordability estimates using actual consumption for the overall population in 2014 is 4.6% which is very much line with the other study. (NB in the paper on p.16 we report the version of this affordability that covers just the rural area, which is 6.4%).**

**Our study specifically targets rural and urban smoker households, both of which will allocate a much higher proportion of their budgets to tobacco products compared to the general population. Socio-economic factors, such as lower incomes and higher smoking prevalence in rural areas, contribute to the higher expenditure on tobacco products for such groups. Furthermore, it is not unusual for studies focusing on particular subgroups, such as heavy smokers or rural households, to report higher tobacco-related expenditures for such groups relative to national averages. See for example: Rossouw, L. (2022) looking at the inequalities of socioeconomic status and tobacco consumption in China, Ghana, India, Mexico, the Russian Federation and South Africa; and Pahari, S. et al. (2023) looking at the meta-analysis of evidence drawn from regional studies of tobacco usage in India between 2010 and 2022.**

**As you rightly observed, the discussion section of our paper interprets these consumption measures in the context of changes in household income and consumption. The high HEPC values reported are an important finding, as they emphasise the economic burden of tobacco consumption on vulnerable subpopulations, particularly in rural areas. Although the 40.5% figure is a startling level to report, we believe it accurately captures the financial strain experienced by these households.**

**In order to recognise the above we have revised the paper in the discussion section (p.15) so that it now reads as:**

*“When we employed 2000RIP with HIPC or HEPC as alternatives to GDP for measuring affordability changes in both rural and urban areas, the outcomes were noticeably different. Indeed, these alternate measures suggest that the amount of financial wealth required is notably larger than is suggested by standard versions of the RIP using GDP. Furthermore, the shift in affordability became more pronounced when we integrated the ConsumptionRIP with HIPC and HEPC.”*

**Furthermore, we have also adjusted the discussion later on (P.16) where we refer the reader to the appendix with population level estimates, in order to highlight why the results in the main paper look considerably different to previously published studies in the first instance. In doing so we explicitly cite the paper from Tan et al. highlighted by the referee. This section now reads as:**

*“When looking the affordability as calculated using mean tobacco use for the entire country population (see S6), we find a relatively lower level of the RIP. This suggests that cigarettes are more affordable when considering the overall population, in contrast to calculations based solely on the smokers’ population. For instance, our ConsumptionRIP-HEPC-Rural, when applied to the overall population, revealed that the percentage of expenditure required to purchase cigarettes was 6.4% in 2014, compared to 43.8% within the smokers’ population. By 2016, these figures had increased to 6.9% and 47.3% respectively. Such differences explain why the results herein might look considerably different from previously published studies on first inspection (see for example, the results in a paper by Tan et al. (2020), which measured the budget share of tobacco products in Malaysia using the Household Expenditure Surveys (HES) and found that the overall budget share for tobacco products was 4.6%)(37). Nevertheless, when considering the changes in the two affordability measures between dates, the aforementioned illustration shows there is broad consistently between the measures. Between 2014 and 2016 there is a change in affordability of 7.8% (6.4% to 6.9%) using the overall population but 7.99% within the smokers’ population (43.8% to 47.3%).”*
